# The Arabidopsis TRM61/TRM6 complex is a *bona fide* tRNA N^1^-methyladenosine methyltransferase

**DOI:** 10.1093/jxb/eraa100

**Published:** 2020-02-25

**Authors:** Jun Tang, Pengfei Jia, Peiyong Xin, Jinfang Chu, Dong-Qiao Shi, Wei-Cai Yang

**Affiliations:** 1 State Key Laboratory of Molecular Developmental Biology, Institute of Genetics and Developmental Biology, Chinese Academy of Sciences, Beijing, China; 2 National Centre for Plant Gene Research (Beijing), Institute of Genetics and Developmental Biology, Chinese Academy of Sciences, Beijing, China; 3 The University of Chinese Academy of Sciences, Beijing, China; 4 Swedish University of Agricultural Sciences, Sweden

**Keywords:** Arabidopsis, AtTRM6, AtTRM61, embryo, N1-methyladenosine, tRNA

## Abstract

tRNA molecules, which contain the most abundant post-transcriptional modifications, are crucial for proper gene expression and protein biosynthesis. Methylation at N^1^ of adenosine 58 (A58) is critical for maintaining the stability of initiator methionyl-tRNA (tRNAi^Met^) in bacterial, archaeal, and eukaryotic tRNAs. However, although research has been conducted in yeast and mammals, it remains unclear how A58 in plant tRNAs is modified and involved in development. In this study, we identify the nucleus-localized complex AtTRM61/AtTRM6 in Arabidopsis as tRNA m^1^A58 methyltransferase. Deficiency or a lack of either AtTRM61 or AtTRM6 leads to embryo arrest and seed abortion. The tRNA m^1^A level decreases in conditionally complemented *Attrm61/LEC1pro::AtTRM61* plants and this is accompanied by reduced levels of tRNAi^Met^, indicating the importance of the tRNA m^1^A modification for tRNAi^Met^ stability. Taken together, our results demonstrate that tRNA m^1^A58 modification is necessary for tRNAi^Met^ stability and is required for embryo development in Arabidopsis.

## Teaser

AtTRM61/AtTRM6 forms a nucleus-localized complex in Arabidopsis that acts as a tRNA m^1^A58 methyltransferase and is essential for embryogenesis

## Introduction

Transfer RNA (tRNA) molecules, typically 76–90 nt in length, are responsible for delivering specified amino acids encoded by messenger RNAs (mRNAs) to the protein synthesis machineries in the cytoplasm and organelles. tRNAs therefore play an essential role in gene expression and protein biosynthesis. Pre-tRNAs undergo a variety of post-transcriptional modifications related to their maturation, stability, folding, and functioning (Helm *et al.*, 1998; Hopper and Phizicky, 2003; Motorin and Helm, 2010; Machnicka *et al.*, 2013). At least 92 chemical modifications have been identified on tRNAs (Machnicka *et al.*, 2013). The functional roles of these modifications are dependent on their chemical properties and on the site on the clover-leaf structure of tRNAs (Burgess *et al.*, 2016). In higher plants, as in bacteria and yeast, tRNAs are post-transcriptionally modified with numerous and diverse chemical moieties. A total of 26 RNA modifications have been identified by a combination of chromatography and mass spectroscopy techniques on purified tRNAs in Arabidopsis and hybrid aspen (Chen *et al.*, 2010; Hienzsch *et al.*, 2013). Among the tRNA nucleoside modifications, methylation is the most prevalent and abundant type (Hori, 2014). The existing data indicate that the modified tRNAs are not only essential for translation, but that they also functional as ‘biosensors’ for plants to adapt to their environmental and physical status (Björk and Neidhardt, 1975; Gustilo *et al.*, 2008; Wang *et al.*, 2017). In the model plants Arabidopsis and rice, the Am, Cm, m^1^A, and m^7^G methylated nucleosides of tRNAs are important for stress responses, while Gm, m^5^U, and m^5^C are involved in development (Chen *et al.*, 2010; Wang *et al.*, 2017). AtTRM5a (At3g56120) catalyses the formation of 1-methylguanosine (m^1^G) and 1-methylinosine (m^1^I) at position 37 on tRNAs and is necessary for vegetative and reproductive growth (Jin *et al.*, 2019). TRM4B (At2g22400) acts as a tRNA 5-methylcytosine (m^5^C) methyltransferase and is required for root development (Burgess *et al.*, 2015; David *et al.*, 2017). AtTRM7 (At5g01230), a 2´-O-ribose methyltransferase, is functional in the efficient immune response to *Pseudomonas syringae* (Ramirez *et al*., 2015). AtTAD2 (At1g48175) and AtTAD3 (At5g24670) catalyse adenosine-to-inosine editing at position 34 of several cytosolic (cyt) tRNA species, and knockout of either gene leads to arrested embryo development at the globular stage (Zhou *et al.*, 2014).

RNA N^1^-methyladenosine is an ancient modification that is conserved throughout archaea, bacteria, and eukaryotes. The free base, 1-methyladenosine (m^1^A), was first discovered by Dunn (1961) and was later found to be present in tRNAs (RajBhandary *et al.*, 1966), rRNAs (Peifer *et al.*, 2013; Sharma *et al.*, 2013), and mRNAs (Dominissini *et al.*, 2016; Li *et al.*, 2016, 2017). tRNA is an indispensable participant in protein synthesis; however, research on the enzymes and regulation involved in the modification of tRNAs is at an early stage, especially in plants (Burgess *et al.*, 2016). Most current knowledge about tRNA m^1^A is derived from archaea, bacteria, and eukaryotes other than plants. The m^1^A nucleoside bears a positive electrostatic charge under physiological conditions, suggesting that it is critical for the structural stability of tRNA through an electro-chemical interaction (Agris, 1996). In (cyt)tRNAs, the m^1^A modification occurs at positions 9, 14, 22, 57, and 58, and positions 9 and 58 have also been reported in mitochondrial (mt) tRNAs (Jühling *et al*., 2009; Suzuki *et al.*, 2011; Oerum *et al.*, 2017). The m^1^A modification at position 58 (m^1^A58) in the TΨC-arm is the most conserved and common in bacteria, archaea, and eukatyotes (Baker, 1971): m^1^A14 is rare and is only found in (cyt)tRNA^Phe^ of mammals, m^1^A22 occurs only in tRNAs from bacteria (Roovers *et al.*, 2008), m^1^A57 just exists as an intermediate to 1-methylinosine (m^1^I) by hydrolytic deamination in archaea (Grosjean *et al.*, 1996), and m^1^A9 has been found in (cyt)tRNA from archaea and mammalian (mt)tRNAs (Helm and Attardi, 2004).

S-adenosyl-L-methionine (AdoMet)-dependent tRNA methyltransferase is responsible for the m^1^A58 modification of tRNA and it has been identified in several organisms. In yeast, two essential proteins, namely GCD14 (TRM61) and GCD10 (TRM6), work in cooperation as tRNA m^1^A58 methyltransferase (Anderson *et al.*, 2000). Sequence and architecture analyses have shown that TRM61 and TRM6 share a common ancestor and were produced by gene duplication and divergent evolution (Bujnicki, 2001; Wang *et al.*, 2016). TRM61 contains a methyl donor (S-adenosyl-L-methionine, SAM) binding pocket and functions as the catalytic subunit, whilst TRM6 is short of the conserved motifs for SAM binding and is essential for tRNA binding. The TRM6 and TRM61 heterodimer forms an L-shaped tRNA-binding region (Wang *et al.*, 2016), and two TRM6-TRM61 heterodimers assemble as a functional heterotetramer (Anderson *et al.*, 2000). TRMI, which is the homolog of yeast TRM61, has been identified as the tRNA m^1^A58 methyltransferase of archaeal and bacterial, and forms a homotetramer to catalyse m^1^A58 modification (Droogmans, 2003; Roovers *et al.*, 2004). Human hTRM61 (TRMT61A) and hTRM6 (TRMT6) also form a complex as (cyt)tRNA m^1^A58 methyltransferase (Ozanick *et al.*, 2005). In the mitochondria of human cells, m^1^A58 modification has been found in tRNA^Leu(UUR)^, tRNA^Lys^, and tRNA^Ser(UCN)^. TRMT61B, a homolog of TRMT61A in humans, forms a homo-oligomer (presumably a homotetramer) and functions as the mitochondria-specific tRNA m^1^A58 methyltransferase (Chujo and Suzuki, 2012). In addition, m^1^A is a reversible modification, a trait it has in common with N^6^-methyladenosine (m^6^A) modification. The first reversible modification to be discovered, m^6^A can be erased by two ALKB family proteins, FTO (Fat mass and obesity-associated protein) and ALKBH5 (alkylation repair homologue protein 5) (Jia *et al.*, 2011; Zheng *et al.*, 2013), while m^1^A58 can be erased by ALKBH1 tRNA demethylase (Liu *et al.*, 2016), indicating that tRNA m^1^A58 is a dynamic modification.

Functional studies have shown that the m^1^A58 modification of tRNA is important for cellular activity and development. Inactivation of *TrmI* results in a thermosensitive phenotype in the bacterium *Thermus thermophilus* (Droogmans, 2003). In yeast, deficiency of either subunit of m^1^A58 methyltransferase results in growth arrest and cell death. Insufficiency of either TRM6 or TRM61 eliminates m^1^A58 modification in tRNAs, and leads to the formation of a non-tRNA-like structure for the initiator methionine tRNA (tRNAi^Met^) and a reduction in levels of mature tRNAi^Met^ (Anderson *et al.*, 1998; Anderson and Droogmans, 2005). The lack of m^1^A58 modification causes structural instability of tRNAi^Met^ by disrupting the unique A54–A58 interaction (Sigler, 1975; Schevitz *et al.*, 1979; Basavappa and Sigler, 1991). In mammalian C6 cells, small interfering RNA (siRNA)-mediated depletion of either TRM6 and/or TRM61 causes significant effects on cell growth, cell death, and tRNAi^Met^ levels (Macari *et al.*, 2016). In addition, loss of *ALKBH1* causes embryonic lethality, defects in neural development, and distortion of the sex ratio in mice (Pan *et al.*, 2008; Nordstrand *et al.*, 2010; Ougland *et al.*, 2012).

It has been determined that tRNA m^1^A58 plays important roles in yeast, mammals, and prokaryotes; however, no m^1^A58 methyltransferase has been yet functionally characterized in plants. Here, we demonstrate that the nuclear-localized AtTRM61/AtTRM6 complex in Arabidopsis acts as a *bona fide* tRNA m^1^A58 methyltransferase, and both *AtTRM61* and *AtTRM6* are essential for embryo development. Insufficient m^1^A58 modification of tRNA is consistent with low levels of tRNAi^Met^ in *Attrm61/LEC1pro::AtTRM61* plants, indicating that tRNA m^1^A58 is vital for the accumulation of tRNAi^Met^. Our findings shed light on the biological functions of tRNA m^1^A58 in plants.

## Materials and methods

### Plant material and growth conditions

*Arabidopsis thaliana* plants of ecotypes Columbia (Col-0) and Ts-1 were grown in an air-conditioned room at 22 °C under a 16/8-h light/dark cycle (90 μmol m^–2^ s^–1^). Seeds of T-DNA insertion mutants (*SALK_024680*, *CS351262*) from the Nottingham Arabidopsis Stock Centre (NASC) were in the Columbia background. Seeds were sterilized with 20% bleach for 10 min, then rinsed five times with sterile water, and germinated on Murashige and Skoog (MS) plates with or without antibiotics.

### tRNA isolation and LC-MS analysis

Total RNA was extracted using TRIzol reagent (Invitrogen) according to the manufacturer’s instruction, with an additional DNase I treatment to eliminate DNA contamination. The RNA was then purified using a RNA Clean & Concentrater^TM^ -5 kit (R1016, Zymo Research).

tRNA was isolated from the total RNA by gel electrophoresis using 7.5% PAGE (29:1 acrylamide:bisacrylamide) containing 7 M urea. Bands of 60–90 nt tRNA were cut from the gel, extracted using 0.3 M NH_4_Ac, and precipitated with glycogen and ethanol. The purified tRNA was then hydrolysed to single nucleosides and dephosphorylated in a 50-μl reaction containing 10 U benzonase (Sigma), 0.1 U phosphodiesterase I (US Biological), and 1 U alkaline phosphatase (NEB). The reaction was kept at 37 °C for 3 h, and then the solution of pre-treated nucleosides was de-proteinized using a Sartorius 10 000-Da MWCO spin filter. Analysis of the nucleoside mixtures was performed using an Agilent 6460 QQQ mass spectrometer with an Agilent 1260 HPLC system. The multi-reaction monitoring (MRM) mode was used because of the high selectivity and sensitivity attained when working with parent-to-product ion transitions. The LC-MS data were acquired using the Agilent Qualitative Analysis software. The MRM peaks of each modified nucleoside were extracted and normalized to quantify the tRNA modifications (Yan *et al.*, 2013; Su *et al.*, 2014). Three technical replicates were used.

### Immuno-northern blot analysis

Immuno-northern blot analysis was performed as described previously (Mishima *et al.*, 2015). Briefly, a total RNA solution with an equal volume of RNA loading buffer (NEB) was denatured by heating at 70 °C for 5 min, followed by immediate chilling on ice. Denatured samples were separated using 12% PAGE/8 M urea denaturing gel electrophoresis at 250 V in 0.5× TBE buffer (45 mM Tris, 45 mM borate, and 1 mM EDTA, pH 8.0). Midori Green was used for RNA staining of the gel. For blotting, the separated RNA was transferred onto a Hybond N+ membrane (GE Healthcare) via electroblotting in 0.5× TBE buffer at 300 mA for 60 min. After UV crosslinking, the membrane was blocked with 1% Blocking Reagent (Roche) in TBST (50 mM Tris, 150 mM sodium chloride, and 0.1% Tween 20, pH 7.4) for 1 h at room temperature and then incubated with anti-m^1^A antibodies (diluted 1:2000; D345-3, MBL) (Dominissini *et al.*, 2016) overnight at 4 °C. Following washing three times with TBST, the membranes were incubated with HRP-conjugated goat anti-mouse IgG (1:10 000) in blocking buffer for 1 h at room temperature. The membranes were again washed three times with TBST, then developed with enhanced chemiluminescence (ECL) western blotting substrate (ThermoFisher Scientific) and imaged using a FluorChem imager (Tanon 5200, Bio-tanon, China).

### *In vitro* transcription of tRNAi^Met^ and northern blotting

The genomic tRNAi^Met^ (At2g23020) fragment was amplified by PCR, and the purified product was used as a template for *in vitro* transcription with T7 polymerase (NEB), after which the tRNAi^Met^ transcript was purified using a RNA Clean & Concentrater^TM^ -5 kit (Zymo Research). Northern blotting procedures were performed according to the DIG Application Manual (Roche) using DIG-tRNAi^Met^ as the probe.

### Bioinformatics analysis of TRM61 and TRM6 homologues

The sequences of yeast TRM61 and TRM6 were used as queries to BLAST search homologs in the NCBI database (http://www.ncbi.nlm.nih.gov/). Multiple sequence alignment was performed using Clustal Omega (https://www.ebi.ac.uk/Tools/msa/clustalo/) and aligned sequences were edited with GeneDoc (https://www.softpedia.com/get/Science-CAD/GeneDoc.shtml). A non-rooted Neighbor-joining tree was constructed using the MEGA5 software (https://macdownload.informer.com/mega-5/).

### Yeast complementation assays

The following yeast strains were obtained from EUROSCARF (www.euroscarf.de): BY4741 (S288C isogenic yeast strain: MATa; his3D1; leu2D0; met15D0; ura3D0), *trm6-506* (BY4741; MATa; ura3Δ0; leu2Δ0; his3Δ1; met15Δ0; gcd10-506:kanMX), and *trm61-4* (BY4741; MATa; ura3Δ0; leu2Δ0; his3Δ1; met15Δ0; gcd14-4:kanMX).

The coding sequences (CDSs) of yeast *TRM61* and *TRM6*, and Arabidopsis *AtTRM6* and *AtTRM61* were amplified and individually cloned with *BamH*I and *Sal*I into pESC-Leu, a vector with the galactose-inducible GAL1-10 promoter active in yeast (Ozanick *et al.*, 2005). To construct the vector for co-expression of *AtTRM6* and *AtTRM61*, the *AtTRM61* CDS was cloned with *Not*I and *Spe*I into pESC-Leu-AtTRM6 to give pESC-Leu-AtTRM6-AtTRM61. The recombinant vectors were transformed into *trm6-506* and *trm14-4*, and the control (empty) vector was transformed into *trm6-506*, *trm14-4*, and BY4741. The transformants were incubated on SD/–Leu plates (synthetic dextrose minimal medium without leucine). Serial dilutions of these incubations were dotted onto selective SCgal/raf plates (synthetic complete media supplemented with galactose and raffinose) at permissive (28 °C) and non-permissive (37 °C) temperatures for 3 d.

### Yeast two-hybrid and co-immunoprecipitation assays

For yeast two-hybrid (Y2H) assays, the CDS fragments of *AtTRM6* and *AtTRM61* were cloned into pGADT7 (AD) and pGBKT7 (BD), respectively (Clontech). The resulting constructs were co-transformed in pairs to yeast strain AH109. The transformed cells were grown on SD/–Leu–Trp (SD/2) and SD/–Trp–Leu–His–Ade (SD/4) plates for 3–7 d at 30 °C.

For co-immunoprecipitation (Co-IP) assays in plant cells, the CDS fragments of *AtTRM61* and *AtTRM6* were cloned into pBSK-35S-C-3×Myc and pBSK-35S-C-3×HA, respectively. Leaf protoplasts were co-transfected with 100 μg plasmid DNA of these constructs and then incubated overnight at 22 °C with 45 rpm shaking. The protoplasts were harvested by soft spinning at 100 *g*, re-suspended in 600 μl native protein extraction buffer [20 mM HEPES-KOH, pH 7.5, 150 mM KCl, 1 mM EDTA, 0.2% Triton-X 100, 1 mM DTT, and protease inhibitor cocktails (Roche)], and vortexed for twice for 1 min to break the cells. The samples were centrifuged at 12 000 *g* for 10 min at 4 °C, the supernatant was collected, and 60 μl was reserved as the control for total protein. The rest was incubated with anti-c-Myc agarose (Sigma) by rotating at 4 °C for 4 h. The samples were spun down at 400 *g* for 3 min and then rinsed five times with washing buffer (154 mM NaCl, 125 mM CaCl_2_, 5 mM KCl, 2 mM MES, pH 5.7), SDS loading buffer was added to samples before western blotting.

### Protein purification and methyltransferase activity assays

The CDSs of *AtTRM61* and *AtTRM6* were amplified and cloned into pET28a^+^ and pGEX-4T-2 plasmids, respectively. For pull-down assays, the constructs pET28a^+^-AtTRM61 and pGEX-4T-2-AtTRM6 were co-transformed into *E. coli*, and induced by 0.5 mM IPTG at 20 °C overnight. Ni-NTA agarose was used to immunoprecipitate the AtTRM61-His/AtTRM6-GST complex. Methyltransferase activity assays of purified Arabidopsis enzyme were conducted as described previously (Ozanick *et al.*, 2005). Briefly, activity assays were carried out at 30 °C for 30 min using 30 mM AdoMet, 150 nM RNA substrate, and 15 nM purified proteins. After the reaction, the RNA substrate was purified and the m^1^A modification level was detected by immuno-northern blotting.

### Phenotypic analysis

To determine the embryo phenotype, siliques were dissected using syringe needles and mounted with Herr’s solution containing lactic acid:chloral hydrate:phenol:clove oil:xylene (2:2:2:2:1, w/w). Embryo development was observed using a Zeiss Axioskop II microscope equipped with differential interference contrast optics.

### Vector construction

For genetic complementation and protein subcellular localization analyses, the *eGFP* tag was fused to the last exon of native *AtTRM61* and *AtTRM6*. For the construction of p1300-*AtTRM61pro::AtTRM61:eGFP* and p1300-*AtTRM6pro::AtTRM6:eGFP*, a 2317-bp fragment upstream ATG was used as the *AtTRM61* promotor, and a 414-bp fragment downstream TAG was used as the terminator. A 741-bp sequence upstream ATG and a 418-bp sequence downstream TGA were used as the *AtTRM6* promotor and terminator, respectively. These constructs were transformed to *Attrm61* and *Attrm6*, respectively.

To construct the CRISPR/Cas9 gene-editing vector, the first exon sequences of *AtTRM61* and *AtTRM6* were each used to search for high-score targets using the software on the CRISPRscan website (http://www.crisprscan.org/?page=sequence). Software on the CRISPR RGEN Tools website (http://www.rgenome.net/cas-offinder/) was used for off-target analysis. Two targets of AtTRM61 (target1 sequence, CCGTCTTTACTTACTTTAACTGG; target2 sequence, GTGTGTATAAGCACTCGGATTGG) and two of AtTRM6 (target1 sequence, TCGCAAGCAAATCTGGGATTTGG; target2 sequence, GTTCTGCTCGACATCAACGATGG) were chosen and cloned into the vector pHEE401E, and these constructs were transformed into Col-0 plants (Xing *et al.*, 2014).

For gene expression analysis, the promotor sequence of *AtTRM61* was cloned into p1300-GUS-3U, and introduced into Col-0.

To construct the conditional complementation vector, p1300-*LEC1pro::AtTRM61*, a 2.7-kb fragment upstream ATG was used as the *LEC1* promotor to drive native *AtTRM61* (from ATG to 414 bp downstream of TAG). p1300-*LEC1pro::AtTRM61* was then transformed into *Attrm61*.

### Protein subcellular localization and gene expression analysis

For examination of protein subcellular localization, the roots of 6-d-old transgenic plants were stained using DAPI. Images of the epidermal cells were captured using a confocal laser scanning microscopy (Zeiss 510 Meta).

For expression analysis, transgenic plants were screened using hygromycin, and 6-d-old whole seedlings, and other parts of flowering plants, were stained with β‐glucuronidase (GUS) as described previously (Chen *et al.*, 2007).

## Results

### Identification of putative genes for tRNA m^1^A58 modification in Arabidopsis

**Fig. 1.**
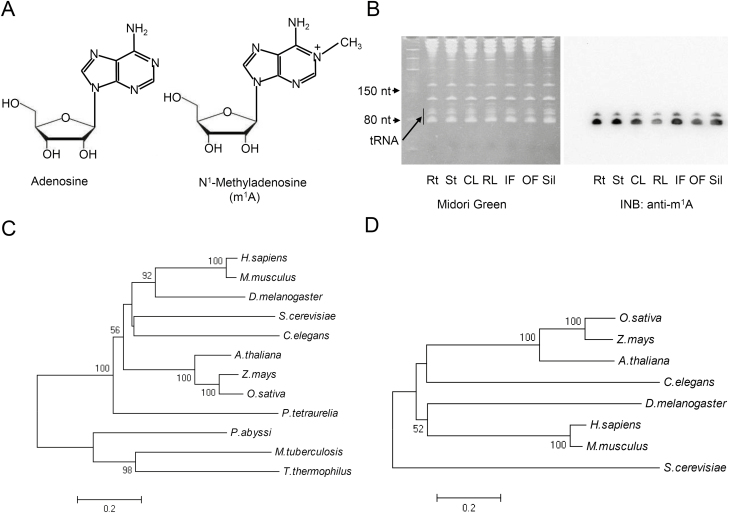
Identification of putative genes for tRNA m^1^A58 modification. (A) Structure of the RNA m^1^A modification. (B) Immuno-northern blotting analysis of the tRNA m^1^A modification in Arabidopsis. Total RNA was isolated from different organs and examined by staining with Midori Green and immuno-Northern blotting using an anti-m^1^A antibody. Rt, roots; St, stems; CL, cauline leaves; RL, rosette leaves; IF, inflorescences; OF, open flowers; Sil, siliques. (C) Unrooted Neighbor-joining tree of TRM61 spanning three Domains, including archaeobacteria (*Thermus thermophilus*, *Pyrococcus abyssi*), eubacterium (*Mycobacterium tuberculosis*), protists (*Paramecium tetraurelia*), fungi (*Saccharomyces cerevisiae*), plants (*A. thaliana*, *Oryza sativa*, *Zea mays*), and animals (*Caenorhabditis elegans*, *Drosophila melanogaster*, *Danio rerio*, *Mus musculus*, *Homo sapiens*). Numbers are values of bootstrap analysis, was performed with 1000 iterations. (D) Phylogenetic tree of TRM6 in Eukarya.

Previous studies using HPLC and LC-MS have indicated that modifications of m^1^A are present on tRNAs of Arabidopsis and rice (Chen *et al.*, 2010; Hienzsch *et al.*, 2013; Wang *et al.*, 2017; Fig. 1A); however, to date no functional studies on plant tRNA m^1^A modification have been conducted. Here, we used LC-MS to confirm the presence of m^1^A modification on tRNAs from 7-d-old seedlings of Arabidopsis (Supplementary Table S1 at *JXB* online). To provide rapid identification of the modification, a method based on RNA m^1^A antibody immuno-northern blotting was adapted to detect and quantify the level of m^1^A. Using this technique, we confirmed that the m^1^A modification was indeed present in tRNAs from different tissues in Arabidopsis (Fig. 1B).

The m^1^A58 modification on tRNAs is catalysed by the TRM61/TRM6 complex in *Saccharomyces cerevisiae* (Anderson *et al.*, 1998). We therefore used the yeast TRM61 and TRM6 protein sequences as queries to BLAST search m^1^A58 candidate genes in the Arabidopsis genome. At5g14600 was the only candidate for yeast TRM61, with a blastP *E*-value of 2×10^–54^ and 41% protein sequence similarity, and it was named as AtTRM61 in TAIR. TRM61 is a conserved protein in archaea, eubacteria, protists, fungi, plants, and animals (Supplementary Fig. S1), but in the phylogenetic tree of the homologs, the plant TRM61s were clearly separated from the other groups (Fig. 1C). Similarly, At2g45730 was the best candidate for the yeast TRM6 homolog in Arabidopsis, with a PSI-BLAST *E*-value of 5×10^–9^ and 28% protein sequence similarity, and it was named as AtTRM6 in TAIR. TRM6 is a conserved protein in Eukarya (Supplementary Fig. S2), and in the phylogenetic tree of eukaryotic TRM6 homologs, animal and yeast TRM6s were separated from those of plants (Fig. 1D).

### *AtTRM61* and *AtTRM6* together rescue yeast m^1^A58-defective mutants

**Fig. 2.**
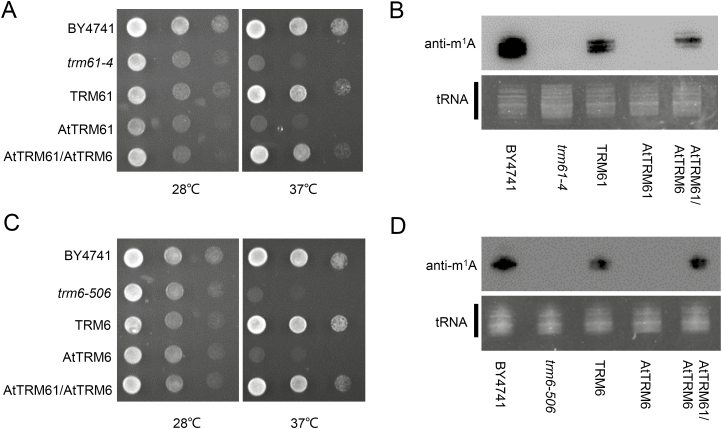
Yeast complementation assays using *AtTRM61* and *AtTRM6*. (A) BY4741 and *trm61-4* cells were transformed with the control vector pESC-Leu, and *trm61-4* cells were also transformed with pESC-Leu carrying *TRM61*, *AtTRM61*, or *AtTRM61*/*AtTRM6*. The transformants were incubated on SD/–Leu plates and then serial dilutions were dotted onto selective SCgal/raf plates at a permissive (28 ℃) or non-permissive (37℃) temperature for 3 d. Each column is a 10-fold dilution. (B) Detection of tRNA m^1^A levels by immuno-northern blotting in the *trm61-4* complement strains. (C) BY4741 and *trm6-506* cells transformed with the control vector pESC-Leu, and *trm6-506* cells complemented with *TRM6*, *AtTRM6*, or *AtTRM61*/*AtTRM6*. (D) Detection of tRNA m^1^A levels by immuno-Northern blotting in the *trm6-506* complement strains.

To investigate whether the candidate genes were true orthologs for TRM61 and TRM6, a yeast complementation assay was employed. The candidate *AtTRM61* and *AtTRM6* genes were introduced into corresponding yeast mutants, *trm61-4* and *trm6-506*, respectively (Supplementary Figs S1, S2). The results showed that cells containing yeast *TRM61*, both *AtTRM61* and *AtTRM6*, and the wild-type control all grew well at 37 °C (Fig. 2A); however, the *trm61-4* mutant cells containing *AtTRM61* alone did not survive. This indicated that *AtTRM61* alone could not rescue the temperature-sensitive phenotype, whilst co-expression of *AtTRM61* and *AtTRM6* could. Similarly, only co-expression of *AtTRM61* and *AtTRM6*, and not *AtTRM6* alone, could rescue the temperature-sensitive phenotype of the *trm6-506* mutant (Fig. 2C). Immuno-northern blotting was also used to further determine whether m^1^A levels in tRNAs were recovered in the transgenic yeast mutants. The m^1^A levels of mutant strains with co-expression of *AtTRM61* and *AtTRM6* were clearly increased, but this was not the case when *AtTRM61* or *AtTRM6* were expressed alone (Fig. 2B, 2D). Since TRM61 and TRM6 function as a heterodimer, it is most likely that AtTRM61 and AtTRM6 were not able to form functional heterodimers with their yeast counterparts, but they were able form a functional heterodimer together in the yeast cells. Similarly, human hTRM6 does not work sufficiently to complement yeast *trm6-504*, and the yeast mutant can only be rescued by co-expression of hTRM6 and hTRM61 (Ozanick *et al.*, 2005). Overall, our data suggested that AtTRM6 and AtTRM61 function as a tRNA m^1^A58 methyltransferase heterodimer in yeast.

### AtTRM61 and AtTRM6 form a complex in Arabidopsis

**Fig. 3.**
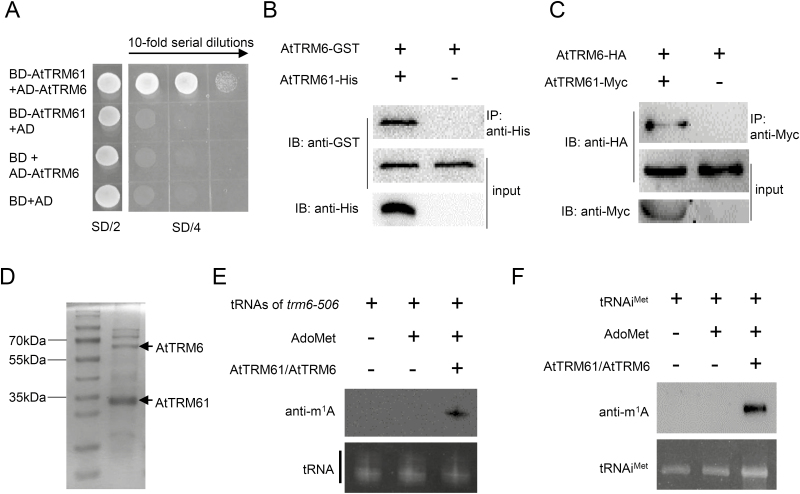
AtTRM61 and AtTRM6 form a complex and show tRNA m^1^A methyltransferase activity *in vitro*. (A) AtTRM61 interacts with AtTRM6 in yeast two-hybrid assays. SD/2, SD/–Leu–Trp; SD/4, SD/–Trp–Leu–His–Ade. (B) AtTRM61 interacts with AtTRM6 in pull-down assays. (**C**) Co-immunoprecipitation assays indicate the interaction between AtTRM61 and AtTRM6 in Arabidopsis protoplasts. (D) SDS-PAGE of the purified AtTRM61-His/AtTRM6-GST complex. (E) tRNA of *trm6-506* was used as the substrate in m^1^A methyltransferase activity assays. AdoMet, S-adenosyl-L-methionine. (F) *In vitro* transcribed tRNAi^Met^ of Arabidopsis was used as the substrate in m^1^A methyltransferase activity assays.

To investigate whether AtTRM61 and AtTRM6 interact in Arabidopsis in the same way that TRM61 and TRM6 function as a heterotetramer to synthesize m^1^A58 in tRNA in yeast, Y2H, pull-down, and Co-IP assays were performed. The results indicated that yeast cells co-expressing BD-AtTRM61 and AD-AtTRM6 grew well on SD/–Trp–Leu–His–Ade selection medium but the control did not (Fig. 3A), which suggested that AtTRM61 interacted with AtTRM6 in yeast. To demonstrate their physical interaction, a pull-down experiment was carried out by co-expression of AtTRM61-His and AtTRM6-GST in *E. coli*, and this indeed showed that AtTRM61-His was able to pull-down AtTRM6-GST (Fig. 3B). These results indicated that AtTRM61 could interact with AtTRM6 *in vitro*. To further verify whether this interaction occurred in Arabidopsis cells, a protoplast Co-IP assay was performed in which AtTRM61-Myc and AtTRM6-HA were co-transformed into leaf protoplasts. The results showed that AtTRM6-HA could be co-immunoprecipitated by AtTRM61-Myc in an anti-c-Myc agarose pull-down assay (Fig. 3C). These data together demonstrated that AtTRM61 could interact with AtTRM6 in Arabidopsis.

### The AtTRM61/AtTRM6 complex has tRNA m^1^A methyltransferase activity *in vitro*

To examine the enzyme activity of the AtTRM61/AtTRM6 complex, AtTRM61-His and AtTRM6-GST were co-expressed in *E. coli* cells and the AtTRM61-His/AtTRM6-GST complex was purified using Ni-NTA agarose (Fig. 3D). The purified complex was then incubated with a methyl donor (SAM) together with a substrate of tRNAs extracted from the yeast mutant *trm6-506* cells, which lacked m^1^A modification. This resulted in m^1^A modification being detected by an anti-m^1^A antibody (Fig. 3E), demonstrating that the AtTRM61/AtTRM6 complex did indeed possess methyltransferase activity. Furthermore, the AtTRM61/AtTRM6 complex was able to catalyse the formation of m^1^A on an *in vitro* transcribed tRNA in Arabidopsis, initiator methionyl-tRNA (tRNAi^Met^, At2g23020) (Fig. 3F). These results confirmed that the AtTRM61/AtTRM6 complex functions as a tRNA m^1^A methyltransferase *in vitro*.

### *AtTRM61* and *AtTRM6* are essential for embryo development

**Fig. 4.**
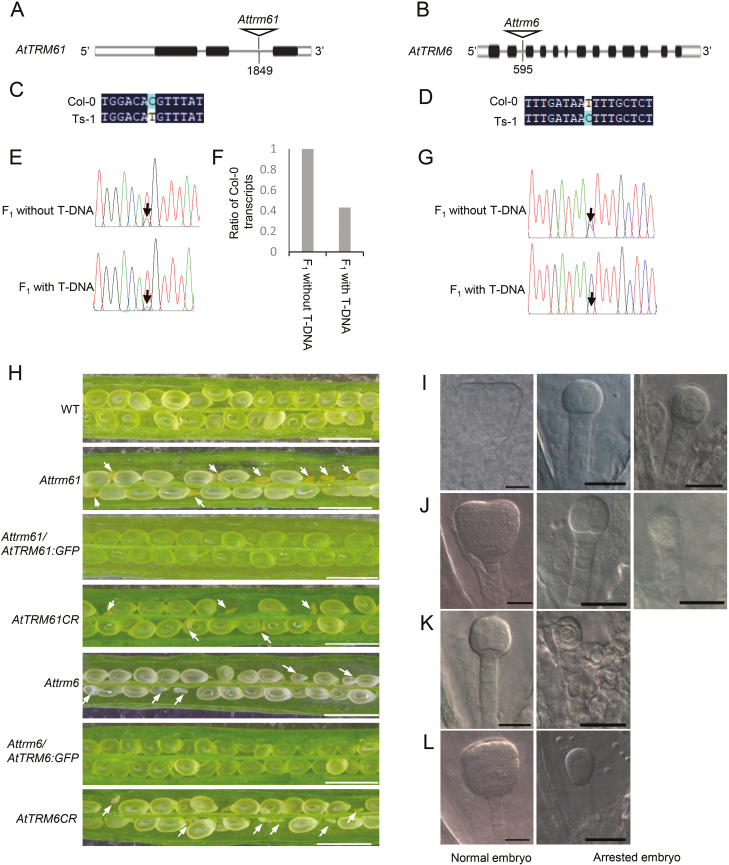
*AtTRM61* and *AtTRM6* are essential for embryo development in Arabidopsis. (A) T-DNA insertion site of *Attrm61* and (B) *Attrm6*. A single-nucleotide polymorphism between Col-0 and Ts-1 in (C) *AtTRM61* and *AtTRM6*(D). (E) Sequencing of *AtTRM61* RT-PCR products from F_1_ plants of the cross *Attrm61*×Ts-1. The arrow indicates transcripts of the Col-0 version. (F) Comparison of the ratio of Col-0 transcripts in F_1_ plants with or without the T-DNA insertion. (G) Sequencing of *AtTRM6* RT-PCR products from F_1_ plants of the cross *Attrm6*×Ts-1. The arrow indicates transcripts of the Col-0 version. (H) Silique phenotype of T-DNA mutants, complemented plants, and CRISPR-Cas9-based knockout plant. Scale bars are 1 mm. Arrows indicate arrested seeds. Normal and arrested embryos in the same siliques of (I) *Attrm61* and (J) *AtTRM61CR* at 4 d after pollination (DAP), and of (K) *Attrm6* and (L) *AtTRM6CR* at 3 DAP. Scale bars in (I–L) are 20 μm.

To study the function of tRNA m^1^A58 modification in Arabidopsis, the T-DNA insertion mutants of *AtTRM61* (Salk_024680) and *AtTRM6* (CS351262) were obtained from NASC and renamed as *Attrm61* and *Attrm6*, respectively. Both mutants contain a T-DNA in the second intron of the gene (Fig. 4A, 4B); however, no homozygous plants could be recovered for either of the mutants (*n>*300 in each case). To investigate whether the insertion affected the targeted gene expression, we made use of the SNPs present in the first exon of *AtTRM61* and in the tenth exon of *AtTRM6* between the Col-0 and Ts-1 ecotypes, respectively (Fig. 4C, 4D). Heterozygous *Attrm61*, and *Attrm6* (Col-0) were crossed with wild-type Ts-1 plants. The full-length CDSs of *AtTRM61* and *AtTRM6* were amplified by RT-PCR from RNAs extracted from F_1_ plants with or without the T-DNA insertion and sequenced. The sequencing signal of *AtTRM61* mRNA in the Col-0 version (*AtTRM61*/Col-0) from plants harboring the T-DNA insert was lower than that of plants without T-DNA (Fig. 4E). The RT-PCR products were subsequently cloned individually to a T-vector and hundreds of colonies were sequenced. In F_1_ plants without T-DNA, the ratio of *AtTRM61/*Col-0 mRNA was 82.4% [C/(C+T), *n*=111]; however, in those plants with the T-DNA insertion, the ratio of *AtTRM61*/Col-0 mRNA was 35.7% (*n*=135). These results indicated that the T-DNA insertion led to a reduction of the level *AtTRM61* mRNA to 43.3% of that of the control (Fig. 4F). This indicated that *Attrm61* is a knockdown mutant. *AtTRM6* mRNA (*AtTRM6*/Col-0) could not be detected in F_1_ plants with the mutation (Fig. 4G), which indicated that *Attrm6* is an *AtTRM6* knockout mutant.

Since no homozygous plants of the *Attrm61* and *Attrm6* mutants were obtained, it was indicative of embryo lethality. Full seed set was observed in wild-type siliques (Fig. 4H), whereas 25.32% (*n*=661) ovules were aborted in *Attrm61* plants (Fig. 4H). Siliques from heterozygous *Attrm61* plants were serially dissected and the ovules were carefully observed after whole-mount clearing with Herr’s solution. In siliques at 4 d after pollination (DAP), the embryos of normal ovules had reached the heart stage of development; however, the embryos of the aborted ovules were arrested between the one- and four-cell stage (Fig. 4I). Similarly, the seed abortion in *Attrm6* heterozygous plants was 27% (*n*=602) (Fig. 4H) and mutant embryos were arrested at the one-cell stage, in contrast to normal embryos that were at the globular stage in the same silique at 3 DAP (Fig. 4K). These data suggested that early embryo development was impaired by knockdown of *AtTRM61* and by knockout of *AtTRM6*.

Genetic complementation assays were performed by introducing constructs of *AtTRM61*-eGFP and *AtTRM6*-eGFP in native regulatory expression boxes to heterozygous *Attrm61* and *Attrm6* plants. Plants homozygous for the *Attrm61* or *Attrm6* mutations with full seed set were obtained (Fig. 4H), confirming that the observed embryo lethality was indeed caused by loss of function of *AtTRM61* or *AtTRM6*. This was further confirmed by CRISPR-Cas9 mutation of either *AtTRM61* or *AtTRM6*. Target sites were chosen on the first exons of *AtTRM61* and *AtTRM6* for construction of the knockout vectors and the constructs were introduced individually into Col-0. Transgenic plants were identified by PCR followed by sequencing, and we obtained eight heterozygous mutants of *AtTRM61* and six of *AtTRM6*. To confirm the mutation type, the PCR products were cloned into a T-vector and sequenced. We found an *AtTRM61* knockout heterozygous plant that contained an adenine insertion after +156 bp of the first exon and an early stop codon at +207 bp (Supplementary Fig. S3A, B). In the T_2_ generation, PCR screening identified a heterozygous plant that was *AtTRM61*-knockout and CRISPR-Cas9 T-DNA-free (hereafter referred to as *AtTRM61CR* plants). The seed set of *AtTRM61CR* plants indicated that the rate of aborted seeds (23%, *n*=425) was consistent with that of the *Attrm61* mutant (Fig. 4H), where embryos were also arrested at the one- to four-cell stage (Fig. 4J). Similarly, in *AtTRM6CR* plants, an adenosine was inserted after +104 bp in the first exon and caused a premature stop codon at position +114 bp (Supplementary Fig. S3C, D). *AtTRM6CR* plants displayed 24.3% (n=1025) seed abortion (Fig. 4H), with embryos arrested at the one-cell stage (Fig. 4L). Taken together, these results demonstrated that *AtTRM61* and *AtTRM6* are essential to embryo development in Arabidopsis.

### AtTRM61 and AtTRM6 are nuclear proteins expressed in fast-growing tissues

**Fig. 5.**
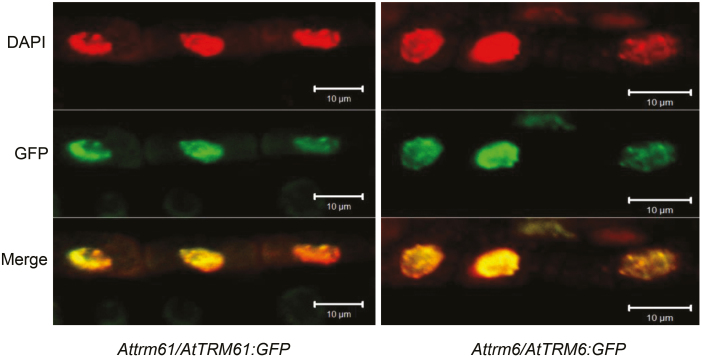
AtTRM61 and AtTRM6 are nuclear proteins. Subcellular localization of AtTRM61 and AtTRM6 in the root cells of 6-d-old seedlings as determined by DAPI staining and green fluorescent protein (GFP) analysis of transformed plants.

Previous studies have shown that yeast TRM61 and TRM6 are both localized in the nucleus (Anderson *et al.*, 1998), whilst human hTRM61 is found in the nucleus and cytoplasm, and hTRM6 is in the nucleus (Macari *et al.*, 2016). To examine the subcellular localization of AtTRM61 and AtTRM6, transgenic plants expressing *AtTRM61::GFP* and *AtTRM6::GFP* were used, and the fluorescent signals were found to be co-localized with the DAPI signal in the nucleus for both constructs (Fig. 5). These results indicated that both AtTRM61 and AtTRM6 are nuclear proteins.

**Fig. 6.**
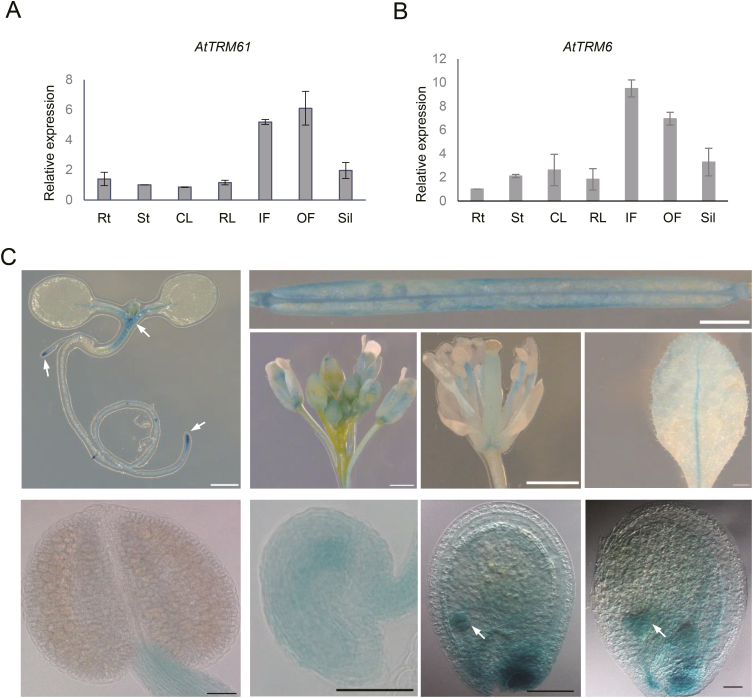
*AtTRM61* and *AtTRM6* are highly expressed in fast-growing tissues of Arabidopsis. Relative expression of (A) *AtTRM61* and (B) *AtTRM6* in different tissues as determined by qRT-PCR. Rt, roots; St, stems; CL, cauline leaves; RL, rosette leaves; IF, inflorescences; OF, open flowers; Sil, siliques. Expression is relative to that of the *ACTIN2* gene. Data are means (±SE) of three replicates. (C) GUS staining of *AtTRM61pro::GUS* plants. Arrows indicate regions where *AtTRM61* was actively expressed. Scale bars are 1 mm in the top row of images and 20 μm in the bottom row.

To investigate the expression patterns of *AtTRM61* and *AtTRM6*, we used qRT-PCR and a GUS reporter system. qRT-PCR analysis indicated that *AtTRM61* and *AtTRM6* transcripts were expressed in vegetative organs such as roots, stems, cauline and rosette leaves, inflorescences, opening flowers, and siliques (Fig. 6A, B). The GUS reporter system indicated that the *AtTRM61* promoter was very active in the fast-dividing cells of the root and shoot tips, inflorescences, flowers, siliques, ovules, and embryos (Fig. 6C). Thus, *AtTRM61* and *AtTRM6* shared similar expression patterns and encoded nuclear proteins, suggesting that they probably play a role in plant growth and development.

### The level of tRNAi^Met^ is correlated with tRNA m^1^A modification

**Fig. 7.**
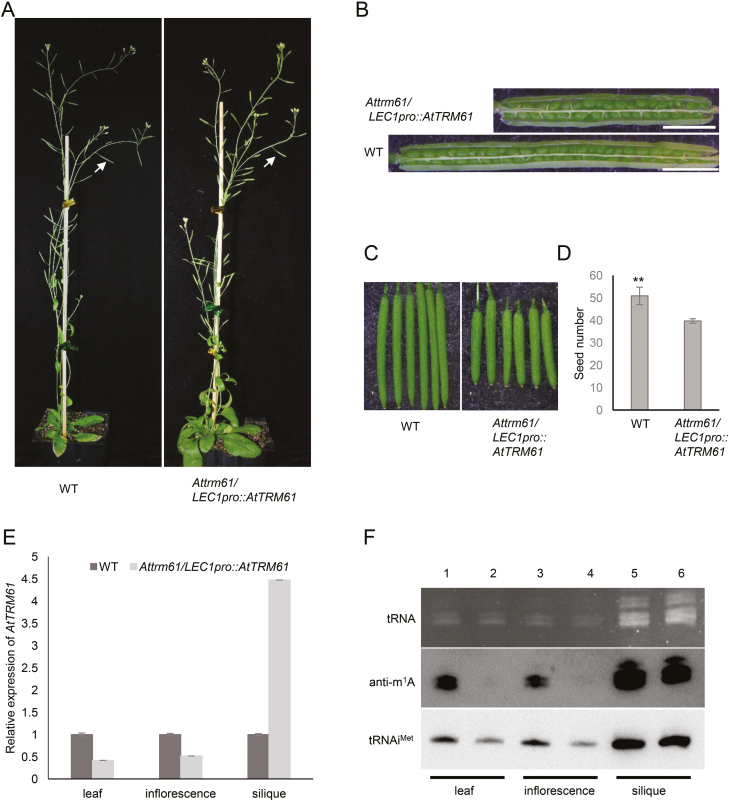
Decreased expression of *AtTRM61* results in reduced tRNA m^1^A modification and decreased levels of tRNAi^Met^. (A) Phenotypes of *Attrm61/LEC1pro::AtTRM61* and the wild-type (WT). Arrows indicate siliques. (B, C) Close-up images of the short siliques of *Attrm61/LEC1pro::AtTRM61*. (D) Seed numbers in *Attrm61/LEC1pro::AtTRM61* and the WT. Data are means (±SE), *n*=30. The significant difference was determined using Student’s *t*-test: **P<0.01. (E) Relative expression of *AtTRM61* in different tissues of the WT and *Attrm61/LEC1pro::AtTRM61*, as determined by qRT-PCR. Expression is relative to that of the *ACTIN2* gene. Data are means (±SE) of three replicates. (F) tRNA m^1^A modification in different tissues as determined by immuno-northern blotting and northern blotting of tRNAi^Met^. Lanes 1, 3, 5 are the WT; Lanes 2, 4, 6 are *Attrm61/ LEC1pro::AtTRM61*.

Given that *AtTRM61* and *AtTRM6* are essential for embryo development, it was not possible for us to obtain a homozygous mutant of either gene. To investigate whether the AtTRM61/AtTRM6 complex was a tRNA m^1^A methyltransferase *in vivo*, we employed a conditional complementation approach. The *AtTRM61* coding region driven by the 2.7-kb promoter of the embryo-specific gene *LEAFY COTYLEDON 1* (*LEC1*) (Lotan *et al.*, 1998), (*LEC1pro::AtTRM61*), was constructed and transformed into *Attrm61* heterozygous plants. A total of 18 transgenic lines were obtained and nine of them gave *Attrm61* homozygous offspring in the T_2_ generation. The homozygous *Attrm61/LEC1pro::AtTRM61* plants bore short siliques (Fig. 7A–D), but they contained normally developed seeds (Fig. 7B). The expression level of *AtTRM61* was restored in the siliques of *Attrm61/LEC1pro::AtTRM61* plants to 4.47-fold that of the wild-type (Fig. 7E), indicating that this transgene was expressed specifically under control of *LEC1pro*. As expected given that *Attrm61* is a knockdown mutant (Fig. 4E, F), the expression of *AtTRM61* in organs other than the siliques was lower than that of the wild-type (42% and 52% compared with the wild-type for leaves and inflorescences, respectively).Correlated with the expression of *AtTRM61*, the level of tRNA m^1^A modification was similar to that of the wild-type in siliques but decreased dramatically in vegetative organs including leaves and inflorescences (Fig. 7F). Interestingly, the m^1^A modification of tRNA was not increased even in siliques that overexpressed *AtTRM61* compared to the wild-type, indicating that overexpression of one component of the AtTRM61/AtTRM6 complex did not correspondingly increase the level of tRNA m^1^A modification. This suggested that AtTRM61 is indeed involved in tRNA m^1^A modification in Arabidopsis cells, and that the AtTRM61/AtTRM6 complex acts as a tRNA m^1^A methyltransferase *in vivo*.

In yeast and mammals, deficiency of tRNA m^1^A58 results in instability of tRNAi^Met^, reduced levels of mature tRNAi^Met^, and interference in the initiation of translation (Anderson *et al.*, 1998; Liu *et al.*, 2016; Macari *et al.*, 2016). We therefore examined the correlation of the abundance of tRNAi^Met^ and tRNA m^1^A levels in different parts of *Attrm61/LEC1pro::AtTRM61* using s northern blotting method. The levels of tRNAi^Met^ deceased in tissues with low levels of tRNA m^1^A, except for very similar levels of tRNAi^Met^ compared to the wild-type in the siliques where the *LEC1* promoter is specifically active, where a normal abundance of tRNA m^1^A was observed (Fig. 7F). These results indicated that the abundance of tRNAi^Met^ was closely associated with the level of tRNA m^1^A modification in Arabidopsis.

## Discussion

In this study, we have shown that the heterodimeric complex AtTRM61/AtTRM6 localized in the nucleus acts as a *bona fide* tRNA m^1^A58 methyltransferase in Arabidopsis. The genes of both proteins were ubiquitously expressed throughout the plant and were essential for embryo development, and knockdown or knockout of *AtTRM61* or *AtTRM6* caused arrest of embryo development at an early stage. The level of tRNAi^Met^ was correlated with tRNA m^1^A modification, with low levels of tRNA m^1^A resulting in reduced tRNAi^Met^, with subsequent disruption of translation of proteins.

Quite a few tRNA modification enzymes are well characterized in *E. coli* and *S. cerevisiae*, and some of them have been to be found fundamental for viability (Persson *et al.*, 1992). In *S. cerevisiae*, there are three enzymes for tRNA modification that are essential, TRM61/TRM6 for m^1^A58 (Anderson *et al.*, 1998), TAD2/TAD3 for adenosine-to-inosine editing at position 34 (Gerber and Keller, 1999), and THG1 for the attachment of a guanine nucleotide to the 5′-end of tRNA^His^ (Gu *et al.*, 2003). In Arabidopsis, it has been shown that the AtTAD2/AtTAD3 complex that edits adenosine-to-inosine at position 34 of several (cyt)tRNA species is indispensable for embryo development (Zhou *et al.*, 2014). I34 at the first position of the anticodon is believed to play a critical role in protein synthesis, because ‘I’ can base-pair with A, C, or U, and this alternative pairing in the third position of codons allows a tRNA to decode multiple codons for the same amino acid (Crick, 1966; Jukes, 1973; Elias and Huang, 2005). However, in Arabidopsis chloroplasts (plastids), a nucleus-encoded tRNA adenosine deaminase arginine (TADA) that is responsible for deamination of the wobble nucleotide of chloroplast tRNA^Arg(ACG)^ to tRNA^Arg(ICG)^ is not necessary for plant survival (Delannoy *et al.*, 2009). Our results showed that another tRNA methyltransferase, the tRNA m^1^A58 methyltransferase complex AtTRM61/ AtTRM6, is essential in Arabidopsis, as it is also necessary in yeast. Knockout of either one of AtTRM61/AtTRM6 caused early embryo arrest (Fig. 4), indicating the key role of the complex in embryogenesis. In yeast and mammals, tRNA m^1^A58 is a major modification and is critical for the stability of tRNAi^Met^, which is vital for the initiation of translation (Liu *et al.*, 2016). Hypomodified tRNAi^Met^ can be polyadenylated by the TRAMP complex and then degraded by exonuclease Rex1p and exosome in yeast (Ozanick *et al.*, 2009). Plant embryo development is characterized by large and rapid amounts of protein synthesis, so the highly dynamic proteome at different stages of embryogenesis will depends on a plentiful supply of tRNAs. The embryo lethality of *Attrm61* or *Attrm6* may have been caused by insufficient tRNAi^Met^ (Fig. 7F), as suggested in yeast (Anderson *et al.*, 2000), or by a general impact on all tRNAs.

tRNAs are highly modified. Cell biology studies have indicated that tRNA modifications are processed via a temporal-special program in the nucleus or cytoplasm. Among the numerous types of modifications, m^1^A58 occurs first, as it takes place on the initial transcripts of tRNA in the nucleus (Hopper, 2013). Our results suggested that the m^1^A58 modification in Arabidopsis was also processed in the nucleus because both AtTRM61 and AtTRM6 were found to be nuclear proteins (Fig. 5). It is not known if the AtTRM61/AtTRM6 complex acts on methylation at other positions of tRNA within the nucleus, but it is not likely that the complex is responsible for modifications generated in the cytoplasm. It is also not conceivable that the AtTRM61/AtTRM6 complex acts in other organelles such as mitochondria or chloroplasts, which encode tRNAs with their own genomes separate from the nucleus (Michaud *et al.*, 2011), since no localization signal was found in the cytoplasm or in these two organelles. It is not known whether m^1^A58 tRNA can be transported from the nucleus to mitochondria or plastids for protein synthesis through subcellular trafficking of tRNA in the nucleus, cytoplasm, and mitochondria (Hopper, 2013; Chatterjee *et al.*, 2018). No studies regarding m^1^A58 modification on mitochondrial or chloroplastic tRNAs in plants have been reported, and the mechanism that underlies this tRNA modification is unknown in these two organelles, although mammalian TRMT6B (a homolog of TRMT61A) has been identified as a mitochondria-specific tRNA m^1^A58 methyltransferase (Chujo and Suzuki, 2012). We therefore implemented a BLASTp search in Arabidopsis for homologs of human TRMT61B, but no candidate other than AtTRM61 showed an *E*-value below 1×10^–6^. Previous phylogenetic analysis has indicated that TRMT61B is of bacterial origin (Chujo and Suzuki, 2012), so we used the protein sequence of TRMI of *T. thermophilus* as a query to search for homologs in Arabidopsis. Apart from AtTRM61, the only other homolog with a BLASTp value below 1×10^–6^ was AtPIMT1 (protein L-isoaspartyl methyltransferase; *E*-value 4×10^–7^). The PIMT enzyme system functions in protein repair and can recognize abnormal L-isoaspartyl residues in polypeptides and convert them to the normal L-aspartyl form. PIMT has consistently been found to be crucial to resistance to ageing, high temperatures, and oxidative stress in bacteria and animals (Mishra and Mahawar, 2019). There are two *PIMT* genes in rice and Arabidopsis, and they are involved in both seed longevity and germination vigor (Xu *et al.*, 2004; Ogé *et al*., 2008; Wei *et al.*, 2015). AtPIMT1 is a cytosol protein, while PIMT2 isoforms are localized to the nucleus (Xu *et al.*, 2004). It would be interesting to determine whether PIMT can methylate tRNAs, and further, whether cytosolic PIMT targets to mitochondria or plastids in Arabidopsis. Recent studies have shown that the TRMT61A/TRMT6 complex is involved in some m^1^A modifications on mRNA in human cells (Li *et al.*, 2017), but whether AtTRM61/AtTRM6 has similar functions in Arabidopsis needs further study.

In summary, we have identified the AtTRM61/AtTRM6 complex as a tRNA m^1^A methyltransferase. Both AtTRM61 and AtTRM6 are nuclear proteins and are required for embryogenesis and protein translation. Our work therefore highlights the importance of tRNA m^1^A modification for embryogenesis in Arabidopsis.

## Supplementary data

Supplementary data are available at *JXB* online.

Fig. S1. Alignment of homologs of TRM61.

Fig. S2. Alignment of homologs of TRM6.

Fig. S3. Mutation site of the knockout plants formed by CRISPR-Cas9.

Table S1. Chemical modifications detected in tRNAs of Arabidopsis as determined by LC-MS.

Table S2. Primers used in this study.

eraa100_suppl_Supplementary_Figures_Tables
