# Supplementary material for: The Arabidopsis TRM61/TRM6 complex is a bona fide tRNA N1-methyladenosine methyltransferase
Source: J Exp Bot. 2020 Feb 25;71(10):3024–36. doi: 10.1093/jxb/eraa100 (PMC7475180; doi:10.1093/jxb/eraa100)
Supplement: eraa100_suppl_Supplementary_Figures_Tables [file eraa100_suppl_supplementary_figures_tables.pdf]

SUPPLEMENTARY INFORMATION

AtTRM61/AtTRM6 complex is a *bona fide* tRNA N^1-methyladenosine methyltransferase in Arabidopsis

Jun Tang[1,3], Pengfei Jia[1], Peiyong Xin[2], Jinfang Chu[2,3], Dong-Qiao Shi[1,3,#], and Wei-Cai Yang[1,3#]

1. State Key Laboratory of Molecular Developmental Biology, Institute of Genetics and Developmental Biology, Chinese Academy of Sciences, East Lincui Road, Beijing 100101, China.
2. National Centre for Plant Gene Research (Beijing), Institute of Genetics and Developmental Biology, Chinese Academy of Sciences, Beijing 100101, China.
3. The University of Chinese Academy of Sciences, Yuquan Road, Beijing 100049, China.

#Authors for correspondence.

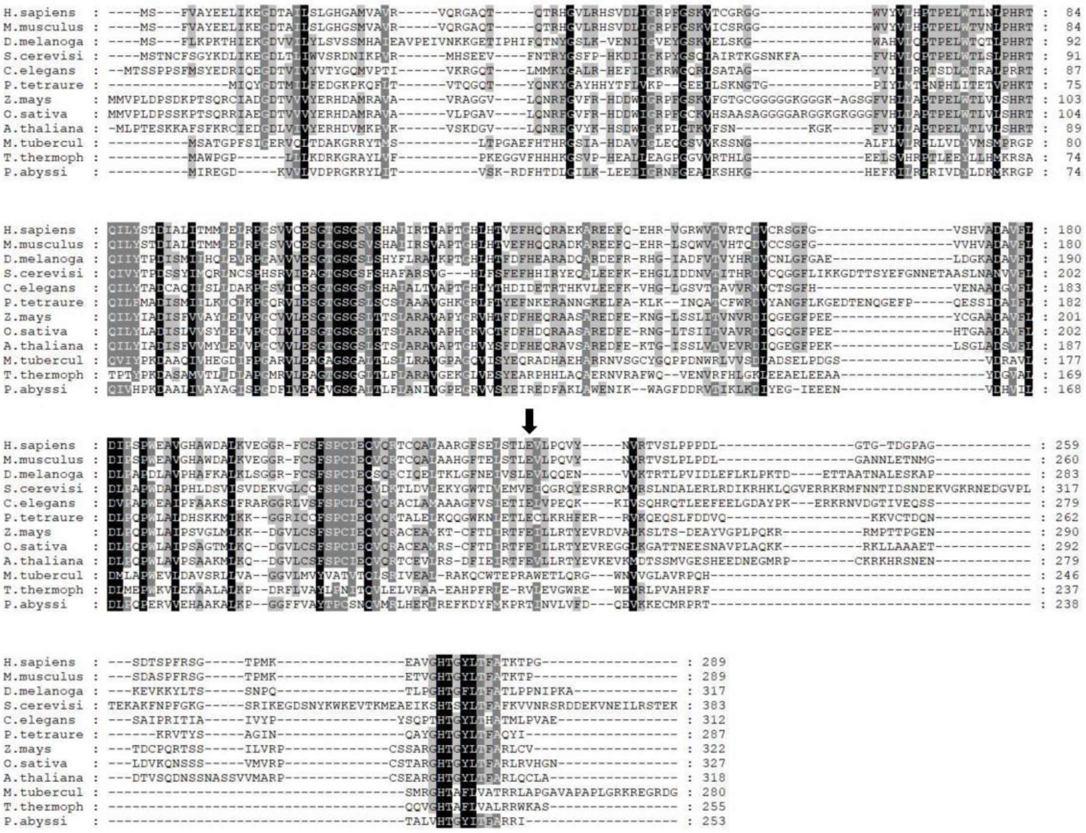

Fig. S1. Homologs alignment of TRM61.

Black arrow indicates a lysine mutated from the wild-type glutamic acid in yeast mutant *trm61-4*.

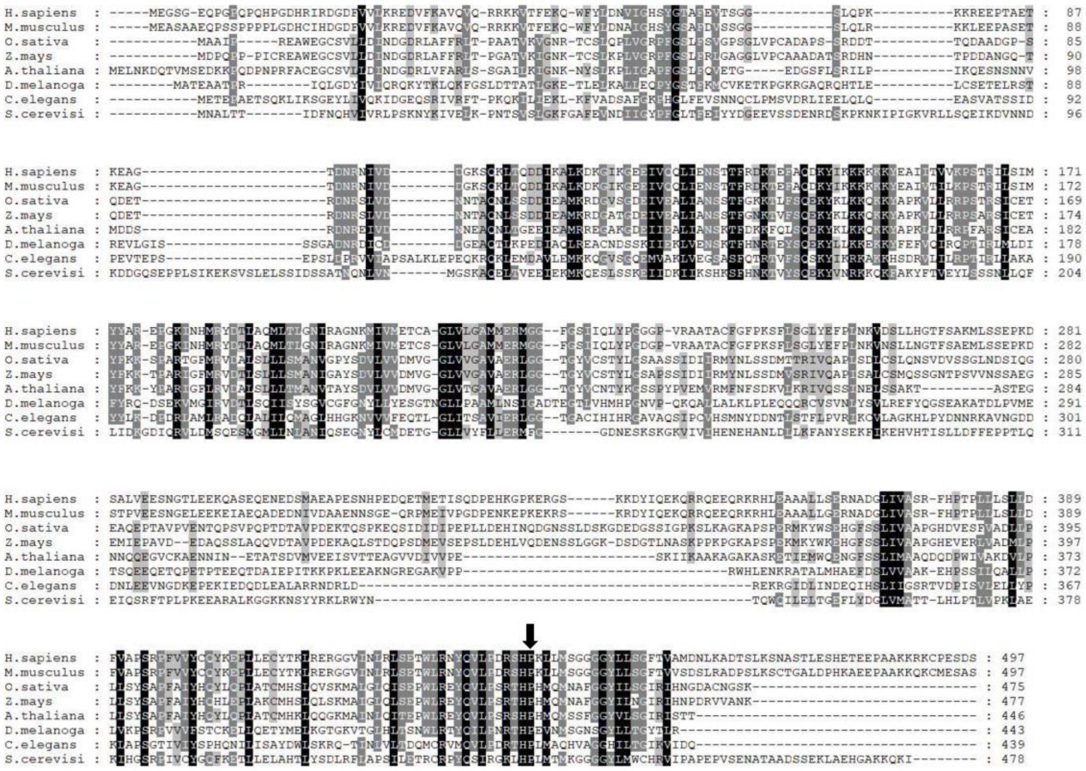

Fig. S2. Homologs alignment of TRM6.

Black arrow shows the mutation from proline to arginine in yeast mutant *trm6-506*.

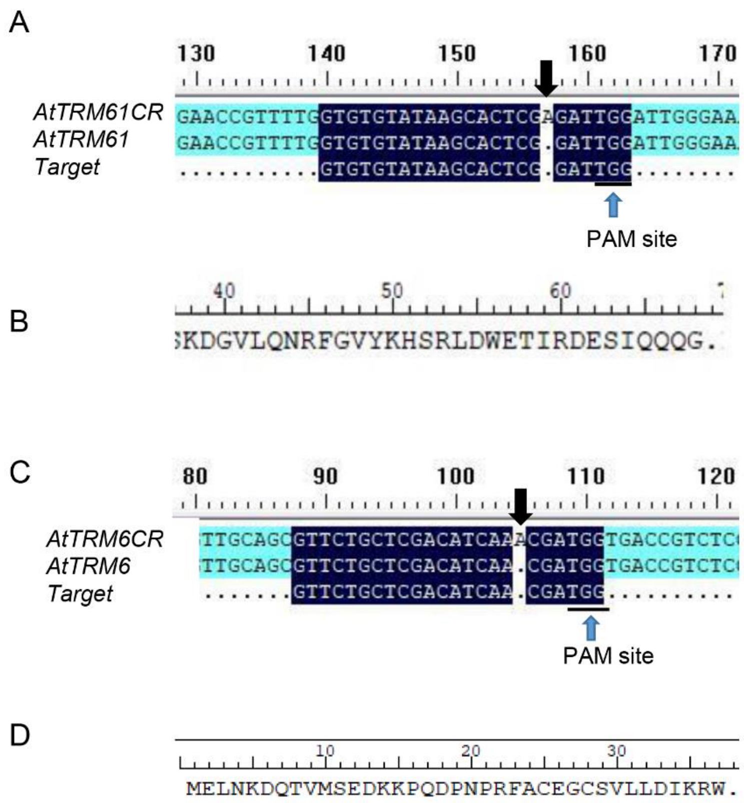

Fig. S3. Mutation sites of the knock-out plants by CRISPR-Cas9.

(A) Gene editting site of *AtTRM61* by CRISPR-Cas9, an A is inserted after position +156bp, black arrow indicated the mutation site. (B) A short ORF was generated by gene edition of *AtTRM61*. (C) Gene editting site of *AtTRM6* by CRISPR-Cas9, an A is insertd after position +104bp, black arrow indicated the mutation site. (D) Gene editting caused a short ORF of *AtTRM6*.

Table S1. Chemical modifications detected by LC-MS in the tRNAs of Arabidopsis

		**Raw Peak Area**			**Normalized Peak Area**		
**Nucleoside**	**Symbol**	**S1-1**	**S1-2**	**S1-3**	**S1-1**	**S1-2**	**S1-3**
cytidine	C	29154849.54	25814250	27941979	29154850	28490117	29749989
adenosine	A	6555042.416	6170370.8	6695646.5	6555042.4	6809982.4	7128894.2
guanosine	G	1388179.906	1186705.1	1365121.1	1388179.9	1309717.2	1453452.5
uridine	U	1168164.492	1027216.2	1129393.9	1168164.5	1133695.9	1202472.3
3'-O-methyladenosine	3'-OMeA	12203.54146	11283.857	13971.68	12203.541	12453.525	14875.73
2'-O-methylcytidine	Cm	274230.6853	361486.75	278238.61	274230.69	398957.94	296242.28
3-methylcytidine	m^3C	3326231.628	3006310.7	3495410.6	3326231.6	3317940.5	3721584.3
5-methylcytidine	m^5C	3326231.628	3006310.7	3495410.6	3326231.6	3317940.5	3721584.3
N^6-isopentenyladenosine	i^6A	33418.68031	25788.895	37693.397	33418.68	28462.134	40132.381
5,2'-O-dimethylcytidine	m^5Cm	ND	ND	ND	ND	ND	ND
1-methyladenosine	m^1A	5192301.817	4793065.4	5233940.4	5192301.8	5289907.5	5572607.1
2-thiocytidine	s^2C	7904.555415	7217.869	7821.1547	7904.5554	7966.0627	8327.2294
N^2, N^2, 7-trimethylguanosine	$m^{2,2,7}G$	2506.713486	2207.6091	2290.3231	2506.7135	2436.4466	2438.5205
N^4-acetyl-2'-O-methylcytidine	ac^4Cm	ND	ND	ND	ND	ND	ND
N6-methyladenosine	m^6A	469633.7833	464358.04	501125.76	469633.78	512492.71	533551.54
3'-O-methylcytidine	3'-OmeC	274230.6853	361486.75	278238.61	274230.69	398957.94	296242.28
2'-O-methyladenosine	Am	12203.54146	11283.857	13971.68	12203.541	12453.525	14875.73
N^2, N^2-dimethylguanosine	$m^2{}_2G$	258518.4854	217272.29	244958.75	258518.49	239794.42	260809.03
5'-O-methylthymidine	5'-OMeT	402.456293	424.43031	514.5723	402.45629	468.42613	547.86815
2'-O-methyluridine	Um	11964.79907	16813.101	13011.606	11964.799	18555.922	13853.533
inosine	I	380371.4606	360899.49	387193.96	380371.46	398309.81	412247.69
2'-O-methylguanosine	Gm	27819.74017	23700.01	27922.901	27819.74	26156.718	29729.676
1-methylguanosine	m^1G	190534.0348	171032.56	192294.28	190534.03	188761.55	204736.85
7-methylguanosine	m^7G	374174.9332	321982.24	334190.5	374174.93	355358.45	355814.59
N^2-methylguanosin	m^2G	190534.0348	171032.56	192294.28	190534.03	188761.55	204736.85
3'-O-methylinosine	3'-OMeI	1066.407763	966.22821	931.80398	1066.4078	1066.386	992.09718
2-thiouridine	s^2U	1099.517154	1663.1474	876.01962	1099.5172	1835.5468	932.70324
4-thiouridine	s^4U	1099.517154	1663.1474	876.01962	1099.5172	1835.5468	932.70324
5-methyluridine	m^5U	188295.1023	163354.97	194047.62	188295.1	180288.1	206603.64
N^4-acetylcytidine	ac^4C	ND	ND	ND	ND	ND	ND
3'-O-methyluridine	3'-OMeU	11964.79907	16813.101	13011.606	11964.799	18555.922	13853.533
5-methyl-2-thiouridine	m^5s^2U	ND	ND	ND	ND	ND	ND
5-methoxyuridine	mo^5U	264.6593981	157.0533	318.13055	264.6594	173.33322	338.71546
pseudouridine	Ψ	5795.452811	5951.7664	5562.6543	5795.4528	6568.7178	5922.5908
2'-O-methylinosine	Im	1066.407763	966.22821	931.80398	1066.4078	1066.386	992.09718
3-methyluridine	m^3U	188295.1023	163354.97	194047.62	188295.1	180288.1	206603.64
1-methylpseudouridine	$m^1\Psi$	9890.805545	9928.5499	9542.3734	9890.8055	10957.729	10159.821
5-hydroxymethylcytidine	hm^5C	8793.649307	8111.0954	7035.1852	8793.6493	8951.8797	7490.4031
5,2'-O-dimethyluridine	m^5Um	ND	ND	ND	ND	ND	ND
hreonylcarbamoyladeno	t^6A	208014.6444	158293.2	193155.23	208014.64	174701.64	205653.51
o-N^6-threonylcarbamoyl	ms^2t^6A	4641.956063	1269.5043	1618.0884	4641.9561	1401.0992	1722.7882
5-carboxymethyluridine	cm5u	131403.3225	111072.38	129741.81	131403.32	122585.98	138136.86

Nucleoside: the name of normal or modified nucleoside detected

Symbol: abbreviation of nucleoside detected

Raw Peak Area: Peak area extracted from LC-MS data

Normalized Peak Area: Peak area normalized to quantity of purified tRNA

ND, Not Detected, signal-to-noise ratio less than 5

m^5C and m^3C could not be separated.

Table S2. Primers used in this study

Primer names	Sequence
LBb1.3	ATTTTGCCGATTTCGGAAC
*attrm61*LP	GTTCCTTCTGCAGCAAAGATG
*attrm61*RP	GGCTTTCGAAGAATGGATCTC
*attrm6*fwd	GAACAAATAAATACCTTCGAGCA
*attrm1*rev	TTACATTTATTTGGGGCAATAGCA
CL20	CTCGTCTTCGCTCGTTTATCTAGT
8474	ATAATAACGCTGCGGACATCTACATTTT
qRT-AtTRM61-F	CAGGATGGAGTTCTATGCTCTTT
qRT-AtTRM61-R	CCAACCATGCTACTGGTATCC
qRT-AtTRM6-F	ATCCATGGGTCTTGGTCTTG
qRT-AtTRM6-R	CTGCGTCTACTGAAGGAAACA
qRT-ACTIN2-F	TACAGTGTCTGGATCGGTGGTT
qRT-ACTIN2-R	CGGCCTTGGAGATCCACAT
AtTRM61GFP-1F	CGACGGCCAGTGCCAAGCTTGAACTATATGATTGAAGAAT
AtTRM61GFP-1R	AGCTAGACATTGGAGTCTTG
AtTRM61GFP-2F	CAAGACTCCAATGTCTAGCTATGGTGAGCAAGGGCGAG
AtTRM61GFP-2R	TTACTTGTACAGCTCGTCCA
AtTRM61GFP-3F	TGGACGAGCTGTACAAGTAATCCAAATTCTCCAACTACAT
AtTRM61GFP-3R	GTACCCGGGGATCCTCTAGAAATTTCTTGATGGATAAAGG
AtTRM61GUS-F	CCTGCAGGTCGACTCTAGAGAACTATATGATTGAAGAAT
AtTRM61GUS-R	ACAGGACGTAACATGGATCCCTTGAGATTATGAAATCACT
AtTRM6GFP-1F	CGACGGCCAGTGCCAAGCTTCTTCTTCTACCTACTAATCA
AtTRM6GFP-1R	AGTGGTAGAGATTCTGATGC
AtTRM6GFP-2F	GCATCAGAATCTCTACCACTATGGTGAGCAAGGGCGAG
AtTRM6GFP-2R	TTACTTGTACAGCTCGTCCA
AtTRM6GFP-3F	TGGACGAGCTGTACAAGTAACTGTTGTTTTCATAACCAGA
AtTRM6GFP-3R	GTACCCGGGGATCCTCTAGAAAGAAAATGAAGCAACGTGG
pBSK-35S-AtTRM61-F	TCGACGGTATCGATAAGCTTATGTTACCAACTGAGTCAAA
pBSK-35S-AtTRM61-R	AGATCTTCTGCAGGAATTCGAGCTAGACATTGGAGTCTTG
pBSK-35S-AtTRM6-F	TCGACGGTATCGATAAGCTTATGGAACTCAATAAGGATCA
pBSK-35S-AtTRM6-R	AGATCTTCTGCAGGAATTCGAGTGGTAGAGATTCTGATGC
pESC-LEU-2-AtTRM6F	GGAGAAAAAACCCCGGATCCATGGAACTCAATAAGGATCA
pESC-LEU-2-AtTRM6R	AACTTCTGTTCCATGTCGACAGTGGTAGAGATTCTGATGC
pESC-LEU-2-AtTRM61F	GGAGAAAAAACCCCGGATCCATGTTACCAACTGAGTCAAA
pESC-LEU-2-AtTRM61R	AACTTCTGTTCCATGTCGACAGCTAGACATTGGAGTCTTG
pESC-LEU-1-AtTRM61F	CCTCACTAAAGGGCGGCCGCATGTTACCAACTGAGTCAAA
pESC-LEU-1-AtTRM61R	TTGTAATCCATCGATACTAGAGCTAGACATTGGAGTCTTG
pESC-LEU-2-AtGCD10F	GGAGAAAAAACCCCGGATCCATGAATGCTTTGACAACCAT
pESC-LEU-2-AtGCD10R	AACTTCTGTTCCATGTCGACTATTTTTTGTTTCTTAGCTC
pESC-LEU-2-AtGCD14F	GGAGAAAAAACCCCGGATCCATGTCAACAAATTGTTTTTC
pESC-LEU-2-AtGCD14R	AACTTCTGTTCCATGTCGACTTTTTCCGTGGATCGAAGAA

BD-AtTRM61-F	TGGCCATGGAGGCCGAATTCATGTTACCAACTGAGTCAA
Primer names	Sequence
AD-AtTRM6-F	CCATGGAGGCCAGTGAATTCATGGAACTCAATAAGGATCA
AD-AtTRM6-R	GATTCATCTGCAGCTCGAgcTCAAGTGGTAGAGATTCTGA
tRNAi^{Met}-INVTRO-F	AGCAGAGTGGCGCAGCGGAA
tRNAi^{Met}-INVTRO-R	TAATACGACTCACTATAGGGAGATGGTAGCAGAGCCAGGTTTC
pGEX-4T-AtTRM6CDS-F	ATCTGGTTCCGCGTGGATCCGAACTCAATAAGGATCAGAC
PGEX-4T-AtTRM6CDS-R	AGTCGACCCGGGGGAATTCCTCAAGTGGTAGAGATTCTGA
pet28a-AtTRM61-F	GGGTCGCGGATCCGAATTCATGTTACCAACTGAGTCAAA
Pet28a-AtTRM61-R	GAGTGCGGCCGCAAGCTTGAGCTAGACATTGGAGTCTTG
CR-AtTRM61DT1-BsF	TAGAGTCGAAGTAGTGATTGCGTCTTTACTTACTTTAACGTT
CR-AtTRM61DT1-F0	TGCGTCTTTACTTACTTTAACGTTTTAGAGCTAGAAATAGC
CR-AtTRM61DT2-R0	AACATCCGAGTGCTTATACACACAATCTCTTAGTCGACTCTAC
CR-AtTRM61DT2-BSR	GCTATTTCTAGCTCTAAAACATCCGAGTGCTTATACACACAA
CR-AtTRM6DT1-BsF	TAGAGTCGAAGTAGTGATTGCGCAAGCAAATCTGGGATTGTT
CR-AtTRM6DT1-F0	TGCGCAAGCAAATCTGGGATTGTTTTAGAGCTAGAAATAGC
CR-AtTRM6DT2-R0	AACTCGTTGATGTCGAGCAGAACAATCTCTTAGTCGACTCTAC
CR-AtTRM6DT2-BSR	GCTATTTCTAGCTCTAAAACTCGTTGATGTCGAGCAGAACAA
LEC1p-AtTRM61pro-F	CGACGGCCAGTGCCAAGCTTCGTCGTTTGCGCCTCCTAGT
LEC1p-AtTRM61pro-R	TGTTTGTGTGCCGTCTTTTT
LEC1p-AtTRM61genome-F	AAAAAGACGGCAGAGAAAACAATGTTACCAACTGAGTCAAA
LEC1p-AtTRM61genome-R	GTACCCGGGGATCCTCTAGAAATTTCTTGATGGATAAAGG
2300-35S-tRNAi^{Met}-F	TTTGGAGAGGACAGGGTACCGTTAAGCGATTATGGATAGT
2300-35S-tRNAi^{Met}-R	CATGCCTGCAGGAACTAGTGGGAAAGCAAACATTTAGTA
